# Supplementary material for: Optimal minimal residual disease threshold in pediatric acute myeloid leukemia: A retrospective cohort study based on the TARGET database
Source: PLoS Med. 2026 May 8;23(5):e1005088. doi: 10.1371/journal.pmed.1005088 (PMC13155632; doi:10.1371/journal.pmed.1005088)
Supplement: S1 Code — (ZIP) [file pmed.1005088.s002.zip › S2 code/PROJ8_16_tbl/PROJ8_16_tbl_seg1_0.pdf]

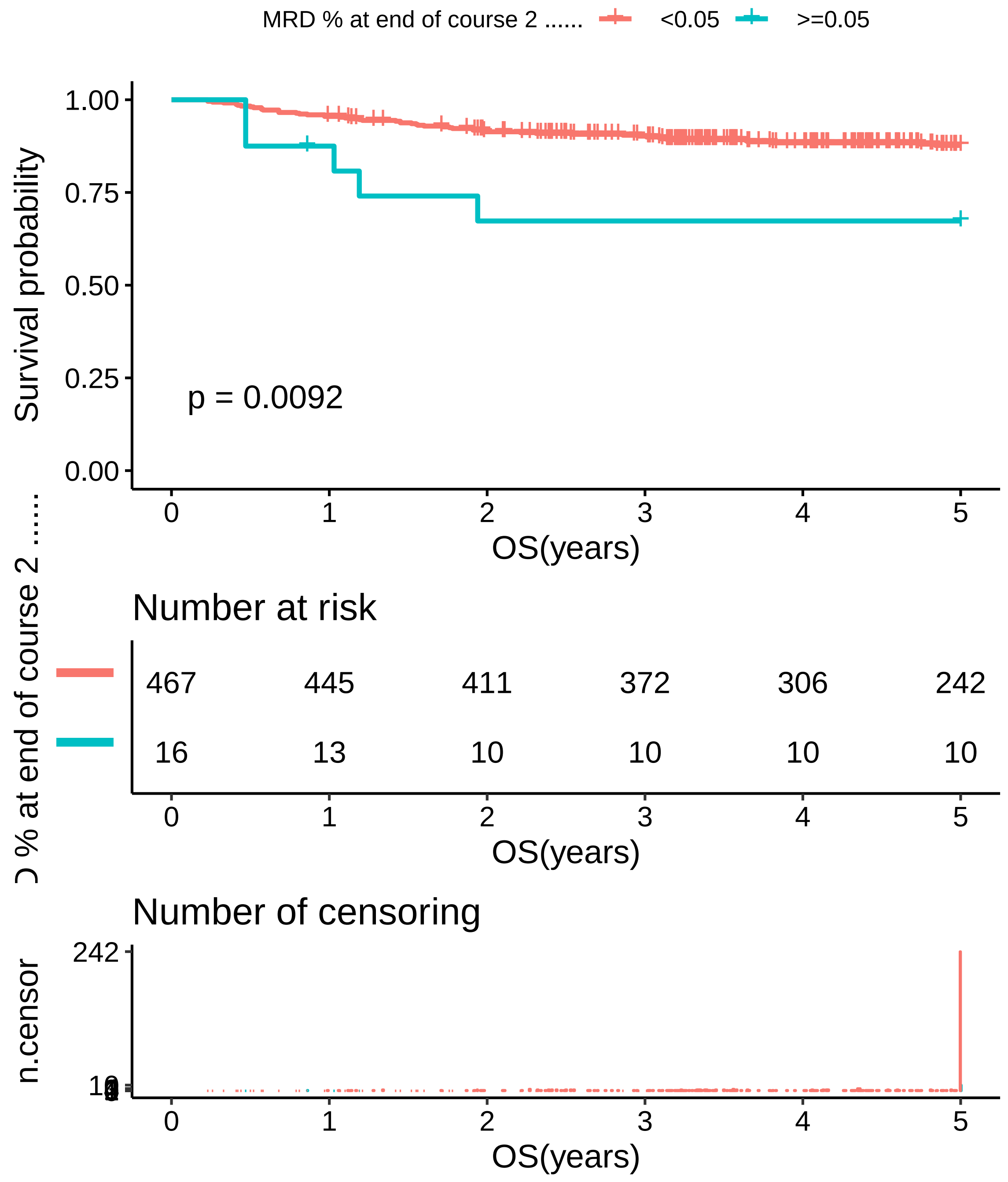

MRD % at end of course 2 ...... <0.05 >=0.05
Survival probability
1.00
0.75
0.50
0.25
0.00
p = 0.0092
0 1 2 3 4 5
OS(years)
Number at risk
MRD % at end of course 2 ......
467 445 411 372 306 242
16 13 10 10 10 10
0 1 2 3 4 5
OS(years)
Number of censoring
n.censor
242
0 1 2 3 4 5
OS(years)
